# Two-year prevalence rates of mental health and substance use disorder diagnoses among repeat arrestees

**DOI:** 10.1186/s40352-020-00126-2

**Published:** 2021-01-07

**Authors:** Lauren A. Magee, J. Dennis Fortenberry, Marc Rosenman, Matthew C. Aalsma, Sami Gharbi, Sarah E. Wiehe

**Affiliations:** 1 O’Neill School of Public and Environmental Affairs, Indiana University Purdue University Indianapolis, 801 W. Michigan St, Indianapolis, IN 46202 USA; 2 Adolescent Medicine, Department of Pediatrics, Indiana University School of Medicine, Indianapolis, IN USA; 3 Department of Pediatrics, Lurie Children’s Hospital, Northwestern University, Chicago, IL USA; 4 Adolescent Behavioral Health Research Program, Department of Pediatrics, Indiana University School of Medicine, Indianapolis, IN USA; 5 Children’s Health Services Research, Department of Pediatrics, Indiana University School of Medicine, Indianapolis, IN USA

**Keywords:** Mental health conditions, Substance use disorder, Repeat arrest, Justice population

## Abstract

**Background:**

Individuals with mental illness and co-occurring substance use disorders often rapidly cycle through the justice system with multiple arrests. Therefore, is it imperative to examine the prevalence of mental health and substance use diagnoses among arrestees and repeat arrestees to identify opportunities for intervention.

**Methods:**

We linked police arrest and clinical care data at the individual level to conduct a retrospective cohort study of all individuals arrested in 2016 in Indianapolis, Indiana. We classified arrestees into three levels: 1 arrest, 2 arrests, or 3 or more arrests. We included data on clinical diagnoses between January 1, 2014 and December 31, 2015 and classified mental health diagnoses and substance use disorder (SUD) based on DSM categories using ICD9/10 diagnoses codes.

**Results:**

Of those arrested in 2016, 18,236 (79.5%) were arrested once, 3167 (13.8%) were arrested twice, and 1536 (6.7%) were arrested three or more times. In the 2 years before the arrest, nearly one-third (31.3%) of arrestees had a mental health diagnosis, and over a quarter (27.7%) of arrestees had an SUD diagnosis. Most of those with a mental health or SUD diagnosis had both (22.5% of all arrestees). Arrestees with multiple mental health (OR 2.68, 95% CI 2.23, 3.23), SUD diagnoses (OR 1.59, 95% CI 1.38, 1,82), or co-occurring conditions (1.72, 95% CI 1.48, 2.01) in the preceding 2 years had higher odds of repeat arrest.

**Conclusions:**

Our findings show that linked clinical and criminal justice data systems identify individuals at risk of repeat arrest and inform opportunities for interventions aimed at low-level offenders with behavioral health needs.

## Introduction

Individuals with mental illness are disproportionately represented in the criminal justice system. Compared to the general population, individuals with mental illness are three times more likely to interact with police and are more likely to be arrested (Hoch, Hartford, Heslop, & Stitt, 2009). Arrestees with mental illness typically receive less than adequate mental health services while incarcerated (Wilper et al., 2009), and many have a co-occurring substance use disorder (Constantine et al., 2010; White, Goldkamp, & Campbell, 2006). Studies have examined serious mental illness among those incarcerated (Baillargeon, Binswanger, Penn, Williams, & Murray, 2009; Constantine et al., 2010; Hwang et al., n.d.), but few have examined mental illness in the general population of arrestees (Becker, Andel, Boaz, & Constantine, 2011), and even fewer have leveraged clinical data to assess diagnoses of mental health and substance use disorder (SUD) among arrestees and repeat arrestees (Constantine et al., 2010). This paper uses arrest data linked to mental health and SUD diagnoses data to describe arrests and subsequent arrests among people with mental health and SUD histories.

Individuals with mental illness tend to cycle through the justice system rapidly (i.e., multiple arrests per year). Prior studies demonstrate repeat arrestees frequent hospital emergency rooms (Akins, Burkhardt, & Lanfear, 2016), are disproportionately homeless (Green, 1997; Harding & Roman, 2017; Tentner et al., 2019), and have co-occurring mental health and SUD diagnoses (White et al., 2006). For instance, nearly half of individuals arrested for a mental health protection hold were rearrested within 60 days, and nearly a quarter of those were rearrested within the first 14 days of release from incarceration (Akins et al., 2016). This work, however, relied on police officers’ perceptions of mental illness (Akins et al., 2016) and not on clinical diagnoses. Furthermore, prior research has relied on different definitions to identify individuals who have frequent contact with the justice system, taking into account only arrests for a protective hold (Akins et al., 2016), or only jail or incarcerated individuals (Baillargeon et al., 2009; Hwang et al., n.d.; Kopak, Guston, Maness, & Hoffmann, 2019; White et al., 2006), or only anecdotal evidence from police departments or news media (Santos & Goode, 2014). Given the limited samples used in prior studies, the true prevalence of mental illness and SUD among repeat arrestees is unknown (Bailey et al., 2018). This is problematic as repeat arrestees are typically presumed to be living with a mental illness (Akins et al., 2016).

One way to improve the identification of individuals with mental illness who have been arrested multiple times is through the use of integrated administrative datasets. A recent study in Chicago used integrated administrative data from the police and fire departments to identify individuals and places most at risk for repeat behavioral health events. The findings demonstrate the utility of large administrative data to identify repeat users and places of emergency services; additionally, these processes can be automated and replicated to assist first responders in resource allocation and in designing proactive interventions (Tentner et al., 2019). Administrative data to identify individuals most at risk for repeat contact with the justice system, along with knowledge of co-occurring mental health and SUD diagnoses, may help guide police when responding to 911 calls.

Police encounters with individuals with mental illness are important to understand for four reasons: (1) encounters may escalate and become violent for all parties involved; (2) encounters are often time consuming for police; (3) arrest is typically the only option police have for individuals in crisis; and (4) identifying opportunities for diversion or other non-incarceration strategies (Reuland, Schwarzefeld, & Draper, 2009; Teplin, 1984). This latter point is frustrating for the responding officers, as arrest perpetuates involvement with the justice system, and arrestees are typically released back into the community without receiving the appropriate clinical or social services. Untreated mental health conditions can lead to recidivism and higher health care and justice system costs (Hwang et al., n.d.; Baillargeon et al., 2009; Regenstein & Rosenbaum, 2014; Reingle Gonzalez & Connell, 2014). Therefore, understanding the prevalence of mental health and SUD diagnoses among repeat arrestees is essential to inform public health strategies on prearrest diversion, implementation of crisis stabilization units, and other social service interventions.

The objectives of this study are to examine the two-year prevalence of mental health and co-occurring SUD diagnoses among arrestees, to examine whether prevalence is higher among those with repeat arrests, and to identify opportunities for intervention for nonviolent offenses. Using population-level justice and clinical care diagnosis data, this study expands prior research by identifying arrestees and repeat arrestees who have clinical diagnoses, not just those who are on a mental health police hold or who have a mental health condition based on a police officer’s perception. This study is guided by the following two hypotheses: (1) repeat arrestees have a higher prevalence than non-repeat arrestees of mental health conditions and SUD; and (2) repeat arrestees for non-violent offenses are more likely than repeat arrestees for violent offenses to have prior mental health conditions and SUD.

## Methods

We conducted a retrospective cohort study of all individuals arrested in Marion County (Indianapolis), Indiana, to assess the associated mental health and SUD diagnoses in the 2 years before arrest. Arrest data were obtained in collaboration with the Indianapolis Metropolitan Police Department (IMPD), which services the majority of Marion County (all of the county except for the independent cities of Lawrence, Speedway, and Beech Grove, and university campuses). Clinical data from the Regenstrief Institute’s the Indiana Network for Patient Care (INPC). The INPC is the largest regional health exchange and data contain more than 17 million patient-level medical records. The INPC was developed over 30 years ago, now is governed as a health information exchange. The health records data have a dual purpose to inform clinical care and to be used for research purposes (Biondich & Grannis, 2004). There is not patient consent for this specific research project but the Indiana University Institutional Review Board approved data access with the protections in place to protect the data. Clinical records include demographic characteristics, encounter dates, and associated diagnosis codes for inpatient, outpatient, and emergency department encounters for all five major hospital systems within Marion County (McDonald et al., 1999). We included all individuals who were arrested between January 1, 2016 and December 31, 2016 (*n* = 22,939) in the cohort; we included a separate indicator of previous arrests that occurred during the period between January 1, 2011 and December 31, 2015. We included data on clinical diagnoses between January 1, 2014 and December 31, 2015.

### Study procedures

We linked individuals from the arrest database to clinical records at an individual level with identifiers including first, middle, and last name, gender, date of birth, social security number, Zip code, and address number. We performed the linkage by using deterministic and probabilistic matching algorithms (Grannis, Overhage, Hui, & McDonald, 2003; Grannis, Overhage, & McDonald, 2002). First, we employed multiple deterministic algorithms that integrate different combinations of identifiers to identify ‘conservative’ matches. Next, we employed multiple probabilistic matching algorithms, which define the probability that a specific pair of data entries is a true match (Grannis et al., 2002; Grannis et al., 2003). Multiple targeted strategies were used to refine the record linkage: we created phonetic transformations using “Soundex” and “NYSISS” methods help link misspelled names; and we matched names with all possible nicknames and known aliases. Three research team members independently reviewed the probabilistic matching algorithms and assigned a score. The most conservative score defined the match. Of the 22,939 arrestees, 21,624 matched to INPC, indicating that 94.3% of individuals who were arrested also had at least one clinical encounter in INPC. Of the 1315 arrestees who did not match: 1243 had 1 arrest and 72 had a repeat arrest. Nonmatched arrestees were more likely to be White individuals and male.

### Measures

Arrestee demographics included race (African American, white, other), sex, and age. We defined mental health diagnoses using ICD-9/10 diagnosis codes at time of any inpatient, outpatient, or emergency department encounter. These were then categorized based on Diagnostic and Statistical Manual (DSM) subgroups (Cauffman, Scholle, Mulvey, & Kelleher, 2005; Lau, Rosenman, Wiehe, Tu, & Aalsma, 2018; Neff, Aalsma, Rosenman, & Wiehe, 2013) to define seven categories for mental health: (1) disruptive behavior disorders, (2) personality disorder, (3) psychosis disorders, (4) anxiety disorder, (5) bipolar disorder, (6) depression, and (7) any other DSM mental health diagnoses. We defined SUD using ICD-9/10 diagnoses codes at time of any clinical encounter type (seeSupplement A for phenotype ICD codes). Multiple mental health diagnoses were defined as having one or more mental health diagnoses within 2014 and 2015. Clinical care utilization was defined as the number of inpatient and/or emergency room encounters where the patient was admitted for any clinical visit, and the number of outpatient encounters for any clinical encounter between 2014 and 2015. We defined three levels of clinical care utilization: (1) individuals with 0 inpatient/ED encounters or outpatient encounters, (2) individuals with 1–3 inpatient/ED encounters or outpatient encounters, and (3) individuals with 4 or more inpatient/ED encounters or outpatient encounters.

For descriptive statistics we defined, frequency of arrests in 2016, in three levels: 1 arrest, 2 arrests, or 3 or more arrests. Arrest history was defined as a binary variable based on any arrest between 2011 and 2015. Arrest types were categorized as follows: (1) violent crime (murder, rape, robbery, aggravated assault), (2) nuisance crime (burglary, larceny, trespassing, prostitution, loitering, littering), (3) immediate detention (protection from self-harm or harm to others), (4) substance related offense (drug or alcohol related offense), (5) motor vehicle offense (prior suspended license, no license ever, other traffic infraction, etc.), or (6) warrant arrest (court ordered, previously committed a crime, or missed a court ordered appointment) (Division, 2000). For multivariable models, we created four separate binary outcome variables; (1) repeat arrests, (2) violent crime, (3) nuisance crime and (4) substance related crime.

### Analysis

We calculated descriptive statistics by using demographic characteristics at the time of each 2016 arrest, except that age for each person was calculated as of 12/31/2016. We calculated two-year prevalence rates for two phenotypes based on diagnoses from any encounter in the period 2014–2015: (1) mental health diagnosis, and (2) SUD diagnosis. We then described the specific mental health diagnoses, clinical care utilization, and arrest type and history.

Lastly, we conducted a multivariable logistic regression to assess the association between mental health and substance use diagnoses and the odds of repeat arrest, controlling for demographics, and clinical care utilization to adjust for access to healthcare (Cook, Trinh, Li, Hou, & Progovac, 2017). Interaction terms were included to examine the relationship between race, mental health and substance use diagnoses and odds of repeat arrest. Analyses were conducted in Stata 10 (StataCorp, 2015).

## Results

In 2016, there were 22,939 individuals with 30,301 arrests and 40,529 charges. Of those arrested, 49% identified as Black individuals, 46% as White individuals, and 4.5% as other. The majority of arrestees were male (71%) and between the ages of 25 and 29 years (Table 1). In the 2 years before the arrest year (2016), 31.3% of arrestees had a mental health diagnosis, and 27.7% of arrestees had an SUD diagnosis. Most individuals with a mental health or SUD diagnosis had both (22.5% of all arrestees) and 25.4% of individuals with a mental health diagnoses had multiple mental health diagnoses. White individuals were more likely to have a mental health or SUD diagnosis compared to Black individuals. Among arrestees with an associated mental health and/or SUD diagnosis, the charges were most often for nuisance or substance related offenses.

**Table 1:** Cohort Descriptive Statistics; Two-year prevalence rates of mental health (MH) and substance use disorder (SUD) among 2016 arrestees in Indianapolis, Indiana

Measures	All Arrestees	Mental Health Only	SUD Only	MH&SUD	Multiple MH Diagnoses	No MH or SUD
	*N* = 22,939	*n* = 2011	*n* = 1173	*n* = 5171	*n* = 5833	*n* = 14,584
	n (%)	n (%)	n (%)	n (%)	n (%)	n (%)
**Race**						
African American	11,319 (49.3)	914 (45.5)	367 (31.3)	2147 (41.5)	2365 (40.6)	7891 (54.1)
White	10,596 (46.2)	1031 (51.3)	781 (66.6)	2707 (52.4)	3127 (53.6)	6077 (41.7)
Other	1024 (4.5)	26 (1.29)	8 (0.68)	134 (2.59)	142 (2.43)	231 (1.58)
Unknown	625 (2.72)	40 (1.99)	17 (1.45)	183 (3.54)	199 (3.41)	385 (2.64)
**Sex**						
Male	16,288 (71.0)	1126 (56.0)	716 (61.0)	3992 (77.2)	4279 (73.4)	10,455 (71.7)
Female	6650 (29.0)	885 (44.0)	457 (39.0)	1179 (22.8)	1554 (26.6)	4129 (28.3)
**Age**						
< 18	482 (2.1)	136 (6.8)	3 (0.3)	79 (1.5)	162 (2.78)	265 (1.82)
18–24	3522 (15.4)	380 (18.9)	114 (9.7)	687 (13.4)	846 (14.5)	2341 (16.1)
25–29	4048 (17.7)	249 (12.4)	214 (18.2)	876 (17.1)	943 (16.2)	2709 (18.6)
30–34	3716 (16.2)	226 (11.2)	251 (21.4)	851 (16.6)	888 (15.2)	2388 (16.4)
35–39	2901 (12.7)	204 (10.1)	209 (17.8)	657 (12.8)	730 (12.5)	1831 (12.6)
40–49	3983 (17.4)	325 (16.2)	217 (18.5)	994 (19.4)	1089 (18.7)	2447 (16.8)
50 +	4248 (18.6)	491 (24.4)	165 (14.1)	992 (19.3)	1140 (19.5)	2600 (17.8)
Unknown	38 (0.17)	0 (0.00)	0 (0.00)	35 (0.68)	35 (0.60)	3 (0.02)
**Prior Criminal Arrest**						
Yes	12,788 (44.3)	1150 (57.2)	886 (75.5)	2893 (55.9)	2570 (44.1)	8474 (58.1)
No	10,151 (55.8)	861 (42.8)	287 (24.5)	2278 (44.1)	3263 (55.9)	6110 (41.9)
**Clinical Utilization** Mean (IQR)						
# inpatient & ED encounters	3.01 (0,1,23)	4.8 (0, 3, 31)	5.1 (0,4,24)	6.7 (0,3,55)	6.2 (1, 3, 7)	2.0 (0, 1, 3)
# outpatient encounters	3.47 (0,1,33)	8.9 (0,6,43)	3.98 (0,2,31)	9.0 (0,5,51)	8.8 (1, 5, 12)	1.8 (0, 0, 2)

**Table 2:** Two-year prevalence rates of mental health (MH) and substance use disorders (SUD), by 2016 arrest prevalence in Indianapolis, Indiana

Measures	1 Arrest	2 Arrests	3 + Arrests
***N*** **= 22,939**	*n* = 18,236	*n* = 3167	*n* = 1536
**Clinical Diagnoses**			
Only MH diagnosis	1538 (8.4)	312 (9.9)	161 (10.5)
Only SUD diagnosis	803 (4.4)	219 (6.9)	151 (9.8)
MH&SUD diagnosis	4241 (23.3)	582 (18.4)	348 (22.7)
Psychosis diagnosis	3549 (19.5)	403 (12.7)	228 (14.8)
Bipolar diagnosis	3649 (20.0)	396 (12.5)	185 (12.0)
Depression diagnosis	4493 (24.6)	551 (17.4)	264 (17.2)
Anxiety diagnosis	4185 (23.0)	474 (15.0)	208 (13.5)
Disruptive behavior disorder diagnosis	3470 (19.0)	380 (12.0)	139 (9.0)
Personality disorder diagnosis	3332 (18.3)	337 (10.6)	126 (8.2)
**Current Crime Type**			
Violent Crime	2660 (14.6)	705 (22.3)	374 (24.3)
Substance use offense	5017 (27.5)	1198 (37.8)	852 (55.4)
Nuisance Crime	3862 (21.2)	1060 (33.7)	884 (57.6)
Immediate Detention	271 (1.5)	64 (2.0)	46 (3.0)
Traffic offenses	3912 (21.5)	807 (25.5)	394 (26.7)
Warrant arrest	3474 (19.5)	1557 (49.2)	1105 (72.0)

Arrest Frequency: Of those who were arrested in 2016, 18,236 (79.5%) were arrested once, 3167 (13.8%) were arrested twice, and 1536 (6.7%) were arrested three or more times. More than three quarters of repeat arrestees were male and Black individuals were re-arrested slightly more than White individuals. Nearly a quarter of individuals with a single arrest had co-occurring mental health and SUD diagnoses, which is slightly higher than the figure of 20% among repeat arrestees (*p* < 0.05). Of individuals with a single arrest, White individuals were more likely than Black individuals to have both a mental health and a SUD diagnosis (*p* < 0.05). Among those with repeat arrests, Black individuals had higher prevalence of psychosis, conduct disorder, personality disorder, and bipolar diagnoses (*p* < 0.05), whereas White individuals had higher prevalence of depression and anxiety disorders (*p* < 0.05). Individuals with repeat arrests most commonly had been charged with nuisance crimes or substance related offenses, or had warrant arrests (*p* < 0.05) (Table 2).

### Multivariable binary logistic regression models

In general, Black individuals and being male had higher odds of repeat arrest. Arrestees with multiple mental health diagnoses, SUD diagnoses, or co-occurring mental health and SUD diagnosis in the preceding 2 years had a higher odds of repeat arrest (*OR 2.68, 95% CI 2.23, 3.23* for mental health; *OR 1.59, 95% CI 1.38, 1.82 for SUD;* *OR 1.72, 95% CI, 1.48, 2.01 for* co-occurring, respectively). Among individuals with a mental health diagnosis, a psychosis diagnosis had a higher odds of repeat arrest (OR 1.34, *95% CI 1.11, 1.61*), whereas, a diagnosis of anxiety (OR 0.36, *95% CI 0.29, 0.43*), personality disorder (OR 0.62, *95% CI 0.47, 0.80*), disruptive behavior disorder (OR 0.56, *95% CI 0.45, 0.71*) or bipolar disorder (OR 0.58, *95% CI 0.48, 0.71*) was associated with lower odds of repeat arrest, respectively (Table 3).

**Table 3:** - Multivariable Binary Logistic Regression Models on Repeat Arrest in 2016, Indianapolis, Indiana

Measure	Model 1	Model 2
	All Arrestees	All Arrestees
	OR (95% CI)	OR (95% CI)
Black individuals	**1.07 (1.00, 1.51)**	**0.81 (0.69, 0.93)**
Male	**1.51 (1.40, 1.64)**	**1.28 (1.15, 1.43)**
Age Group		
< 18	0.87 (0.67, 1.13)	0.88 (0.68, 1.15)
18–24	0.92 (0.82, 1.04)	0.92 (0.82, 1.04)
25–29	reference category	reference category
30–34	1.05 (0.94, 1.17)	1.04 (0.93, 1.16)
35–39	0.98 (0.87, 1.11)	0.97 (0.86, 1.10)
40–49	0.89 (0.79, 0.99)	**0.89 (0.79, 0.99)**
50 +	**0.87 (0.77, 0.96)**	**0.86 (0.76, 0.96)**
Inpatient/Emergency		
No encounters	**0.74 (0.67, 0.81)**	**0.74 (0.67, 0.81)**
1–3 encounters	reference category	reference category
4 or more encounters	**1.37 (1.26, 1.49)**	**1.39 (1.27, 1.51)**
Outpatient		
No encounters	**1.12 (1.03, 1.23)**	**1.12 (1.03, 1.23)**
1–3 encounters	reference category	reference category
4 or more encounters	**0.89 (0.81, 0.99)**	0.91 (0.82, 1.01)
Substance Use disorder only diagnosis	**1.59 (1.38, 1.82)**	**1.74 (1.47, 2.05)**
Mental health and substance use diagnosis	**1.72 (1.48, 2.01)**	**1.61 (1.31, 1.97)**
Multiple mental health diagnosis	**2.68 (2.23, 3.23)**	**3.17 (2.40, 4.18)**
Psychosis diagnosis	**1.34 (1.11, 1.61)**	0.92 (0.68, 1.25)
Bipolar diagnosis	**0.58 (0.48, 0.71)**	**0.58 (0.44, 0.76)**
Depression diagnosis	0.88 (0.76, 1.01)	0.95 (0.79, 1.15)
Anxiety diagnosis	**0.36 (0.29, 0.43)**	**0.33 (0.25, 0.42)**
Disruptive behavior diagnosis	**0.56 (0.45, 0.71)**	**0.65 (0.47, 0.89)**
Personality diagnosis	**0.62 (0.47, 0.80)**	**0.51 (0.35, 0.73)**
Black*Male		**1.43 (1.22, 1.67)**
Black* Substance Use disorder only diagnosis		**0.71 (0.52, 0.94)**
Black* Mental health and substance use diagnosis		1.06 (0.78, 1.44)
Black* Multiple mental health diagnosis		**0.66 (0.45, 0.96)**
Black* Psychosis diagnosis		**1.83 (1.25, 2.68)**
Black* Bipolar diagnosis		1.05 (0.71, 1.56)
Black* Depression diagnosis		0.83 (0.62, 1.10)
Black* Anxiety diagnosis		1.13 (0.77, 1.64)
Black* Disruptive behavior diagnosis		0.90 (0.57, 1.40)
Black*Personality diagnosis		1.55 (0.92, 2.60)

Bold values indicate significant values at a *p* < 0.05 level

To better understand these findings, we included an interaction term for race. Black males have higher odds of repeat arrest (OR 1.43, 95% CI 1.22, 1.67) compared to White males. Black individuals with a psychosis diagnosis in the 2-years before arrest have nearly two-fold higher odds of repeat arrest (OR 1.83, *95% CI 1.25, 2.68*). Compared to White individuals, having multiple mental health diagnosis lowers the odds of repeat arrest (OR 0.66, 95% CI 0.45, 0.96) for Black individuals. Black individuals with a SUD diagnosis have lower odds of repeat arrest compared to White individuals (OR 0.71, 95% CI 0.52, 0.94). No differences between race were observed for bipolar disorder, depression, anxiety, disruptive behavior disorder or personality diagnosis (Table 3).

When examining repeat arrestees and crime type, Black individuals, males, and individuals under 18 years of age had higher odds of repeat arrest for violent crime (OR 1.44, 95% CI 1.23, 1.67; OR 1.46, 95% CI 1.22, 1.76; OR 2.74, 95% CI 1.68, 4.47). Among repeat arrestees, individuals with a SUD diagnosis (OR 0.54, 95% CI 0.38, 0.75) and anxiety diagnosis (OR 0.67, 95% CI 0.48, 0.92) had a lower odds of violent crime arrest (Table 4). As expected, among repeat arrestees, individuals with a SUD diagnosis had increased odds of a substance related arrest (OR 1.40,95% CI 1.04, 1.88). None of the associated mental health or SUD diagnoses were associated with nuisance related arrests.

**Table 4:** Multivariable Binary Logistic Regression on Crime Type, among 2016 repeat arrestees, Indianapolis, Indiana

Measure	Model 1	Model 2	Model 3
*n* = 4580	Outcome = Violent Crime	Outcome = Nuisance Crime	Outcome = Substance related Crime
	OR (95% CI)	OR (95% CI)	OR (95% CI)
Black individuals	**1.44 (1.23, 1.67)**	1.10 (0.95, 1.27)	**0.83 (0.71, 0.97)**
Male	**1.46 (1.22, 1.76)**	0.87 (0.74, 1.03)	**1.29 (1.07, 1.54)**
Age Group			
< 18	**2.74 (1.68, 4.47)**	**0.19 (0.12, 0.31)**	**0.14 (0.05, 0.40)**
18–24	**1.50 (1.17, 1.92)**	**0.45 (0.35, 0.57)**	1.12 (0.86, 1.47)
25–29	reference category	reference category	reference category
30–34	0.82 (0.64, 1.05)	1.07 (0.84, 1.37)	1.09 (0.85, 1.38)
35–39	1.01 (0.77, 1.31)	1.01 (0.78, 1.31)	**1.32 (1.01, 1.72)**
40–49	1.00 (0.78, 1.28)	0.93 (0.73, 1.19)	1.07 (0.84, 1.38)
50 +	1.13 (0.89, 1.44)	**0.75 (0.59, 0.95)**	1.16 (0.90, 1.49)
Inpatient/Emergency			
No encounters	0.91 (0.74, 1.10)	0.95 (0.79, 1.14)	1.08 (0.89, 1.32)
1–3 encounters	reference category	reference category	reference category
4 or more encounters	1.04 (0.87, 1.24)	**1.29 (1.08, 1.53)**	0.97 (0.81, 1.17)
Outpatient			
No encounters	0.95 (0.78, 1.16)	1.07 (0.89,1.28)	**1.29 (1.06, 1.58)**
1–3 encounters	reference category	reference category	reference category
4 or more encounters	**1.39 (1.12, 1.73)**	0.93 (0.76, 1.15)	0.93 (0.74, 1.18)
Substance Use disorder only diagnosis	**0.54 (0.38, 0.75)**	0.97 (0.74, 1.28)	**1.40 (1.04, 1.88)**
Mental health and substance use diagnosis	1.10 (0.71, 1.71)	1.21 (0.82, 1.76)	1.13 (0.73, 1.75)
Multiple mental health diagnosis	**1.71 (1.21, 2.42)**	0.75 (0.53, 1.06)	0.91 (0.59, 1.37)
Psychosis diagnosis	0.75 (0.54, 1.04)	1.14 (0.83, 1.56)	0.91 (0.61, 1.36)
Bipolar diagnosis	1.01 (0.71, 1.44)	1.13 (0.80, 1.59)	0.85 (0.56, 1.28)
Depression diagnosis	0.91 (0.69, 1.21)	0.85 (0.65, 1.11)	0.98 (0.71, 1.35)
Anxiety diagnosis	**0.67 (0.48, 0.92)**	0.90 (0.65, 1.24)	1.17 (0.79, 1.73)
Disruptive behavior diagnosis	1.25 (0.83, 1.87)	1.37 (0.92, 2.05)	0.61 (0.32, 1.02)
Personality diagnosis	1.15 (0.73, 1.81)	0.81 (0.52, 1.27)	1.12 (0.64, 1.99)

Bold values indicate significant values at a *p* < 0.05 level

## Discussion

Our study demonstrates the value of large administrative data sets linked at the individual level in understanding the prevalence of mental health conditions and SUD among repeat arrestees. As expected, 30–40% of arrestees had a mental health or SUD diagnosis, and about 20% had co-occurring mental health and SUD diagnoses. Overall, our findings follow national trends for incarcerated individuals which demonstrate that 26% of jail inmates and 14% of prisoners experienced psychological distress within a month of incarceration (Bronson & Berzofsky, 2017). Additionally, our results suggest individuals with co-occurring mental health and SUD diagnoses have a greater likelihood of repeat arrest, which aligns with prior research on prison inmates (Baillargeon et al., 2009). Our findings regarding mental health diagnosis and repeat arrest, however, differ from prior research. Our findings suggest that anxiety disorder, disruptive behavior disorder, bipolar disorder, depression, and personality disorder are associated with a lower likelihood of repeat arrests. Prior research demonstrated individuals with major depression, bipolar, schizophrenia and other psychotic disorders had an increased risk of repeat incarcerations within a 6-year time period (Baillargeon et al., 2009). These observed differences may be because populations were assessed at different points within the criminal justice system. For instance, most prior research on mental health and repeat recidivism has been conducted on incarcerated individuals (Hwang et al., n.d.; Baillargeon et al., 2009; Kopak et al., 2019; White et al., 2006). Incarcerated individuals are typically sentenced to prison for longer than 1 year and receive mental health and substance use screening upon prison intake (Forrester, Till, Simpson, & Shaw, 2018; Martin, Potter, Crocker, Wells, & Colman, 2016). Our results, however, show the high prevalence of mental health and SUD diagnoses among arrestees and especially among those arrested multiple times in a narrow time frame, who typically have only brief contact with the justice system and receive little or no mental health services compared to those incarcerated for longer periods (Regenstein & Rosenbaum, 2014; Wilper et al., 2009). Yet, these arrests, and repeat arrests, may disrupt their employment, add additional stress, and limit their access to health care services (Wang et al., 2008).

Our study also demonstrates racial disparities in mental health and SUD diagnoses among arrestees. Black individuals represent nearly half of all arrestees, however, White individuals are more likely to have had a mental health or SUD diagnoses in the 2 years prior to arrest and had a higher likelihood of repeat arrest for both nuisance and substance related offenses. Overall, individuals with multiple mental health diagnosis were more likely to experience repeat arrest, however, when race was entered into the model, Black individuals with multiple mental health diagnoses had lower odds of repeat arrest. Black individuals with a psychosis disorder diagnosis were more likely to experience repeat arrest, whereas a SUD diagnosis is related to higher odds of repeat arrest for White individuals. These findings align with prior work which demonstrated that Black individuals were over-represented among prison inmates with psychotic disorders (Baillargeon et al., 2009) and White prison inmates have higher rates of SUD alone compared to Black inmates (Baillargeon et al., 2009).

Differences by mental health disorder in risk of repeat arrest may be explained in how the mental illness or SUD is flagged and is addressed within the criminal justice court process. Our findings demonstrate that arrestees with a personality disorder were less likely to be arrested more than once; however, a psychosis and personality disorder may be identified earlier in the court process, which could result in the individual being assigned to mental health court or being retained in custody. By contrast, our results demonstrate an increased risk of repeat arrest for individuals with SUD, which is usually detected later in the court process, if ever (Arndt, Turvey, & Flaum, 2002; Maschi & Dasarathy, 2019). Understanding the mental health and SUD diagnoses among arrestees at the time of arrest and at the time of the initial court screening may be an opportunity to divert individuals to treatment or to assign other resources within the justice system. However, without linked clinical and police data, diagnoses may not be detected early in the process, or ever.

Understanding behaviors associated with mental health conditions and SUD diagnosis may *also* help improve police-citizen interactions, allow police to deescalate situations, and link individuals to the appropriate services. Over the past 20 years, police departments have taken additional steps to educate officers how best to identify signs of mental illness and to implement techniques to deescalate volatile situations through Crisis Intervention Training (CIT). CIT has decreased arrests and has increased linkages to services (Compton et al., 2014). In addition to CIT, a number of jurisdictions have implemented mental health courts which aim to improve adherence to treatment as a means of avoiding incarceration (Kuehn, 2007); however, these courts are not able to serve all individuals with a mental illness who enter the justice system. More recently, jurisdictions have implemented diversion programs for individuals with mental health (Bailey et al., 2018) conditions or SUD (Collins, Lonczak, & Clifasefi, 2017) who commit low level, non-violent offenses; diversion is a way to link individuals to appropriate services and to medical professionals and has been shown to reduce repeat arrests. Programs like Seattle’s Law Enforcement Assisted Diversion, which diverts individuals into supportive case management programs instead of jail, have reduced subsequent arrests by nearly 60% for individuals involved in low-level drug and prostitution activity (Collins et al., 2017).

Our findings support such diversion programs, as the majority of individuals arrested more than once were arrested for nuisance and substance related offenses. Additionally, among repeat arrestees, those with an SUD diagnosis were more often arrested for substance related offenses. Our results also show that individuals with a co-occurring mental health and SUD diagnosis and multiple mental health diagnosis have a greater likelihood of repeat arrest. However, our findings suggest individuals with a mental diagnosis, other than psychosis, are not at higher odds for repeat arrests, compared to individuals arrested only once. These findings partially support the public narrative about “frequent fliers” within the justice system, which presumes that individuals are repeatedly arrested because of behavioral health conditions (Akins et al., 2016; Harding & Roman, 2017). However, our findings may suggest the “frequent fliers” may well be distinct by the type of mental health disorder (e.g., psychosis versus depression, etc.) and by the severity of mental health (e.g., co-occurring mental health and SUD diagnosis versus anxiety disorder). These differences may be in linkage to medical care or other underlying social factors which increase the frequency of their contact with the police. Programs such as Crisis Stabilization Centers provide behavioral health treatment within the community, thereby, limiting the need for individuals to seek inpatient and emergency department care. Such programs help individuals relieve immediate crisis and have proven cost-effective (Heyland, Emery, & Shattell, 2013; Saxon, Mukherjee, & Thomas, 2018). Additionally, community-wide teams of mental health, social service, educational, clinical care, and other professionals who work together to form social and economic interventions in parallel with mental health services (Maschi & Dasarathy, 2019) may be opportunities to better serve this high-needs population. An example of such a program can be found in Connecticut’s “The Connection” program, which provides behavioral health services, job training, and family and housing support under the same state agency for the state’s most vulnerable populations (Lee, Sells, Hasson, Klimczak, & Barber, 2019).

Lastly, among repeat arrestees, Black individuals and individuals with multiple mental health diagnoses have higher odds of being arrested for a violent crime; however, having a diagnosis for anxiety lowers the likelihood of repeat arrest for violent crime. These findings demonstrate that mental illness is not associated with repeat violent crime arrests in 2016. Nevertheless, the findings highlight racial disparities in the justice population. African Americans disproportionally represent those convicted of a violent crime among the US prison population (Bronson & Berzofsky, 2017); however, other research demonstrates a slight increase in violence for those with a psychosis diagnosis (Baillargeon et al., 2009; Douglas, Guy, & Hart, 2009; Fazel, Gulati, Linsell, Geddes, & Grann, 2009). The difference in the reported findings may be that previous studies have assessed whole prison populations, whereas we examined repeat arrestees. This is a clear direction for future research.

### Limitations

Our study has several limitations. Our study used both arrest and clinical records to examine associated diagnoses among the justice population; however, not all individuals have access to or utilize the clinical care system at the same rate and nearly 6 % of arrestees did not link to INPC data. Therefore, our findings may underestimate the number of arrestees diagnosed with mental health and/or SUD diagnoses. We adjusted as best we could for clinical care utilization but our categories may miss the complexity of clinical care and mental health and this is a clear direction for future research. Our findings also may speak to a larger issue of a lack of clinical care based on socioeconomic barriers, lack of transportation, or other social confounding factors. We included interaction terms on race to determine disparities in diagnoses and arrests; however, we were unable to examine ethnicity due to incomplete and unreliable data for that variable. We extended prior studies to examine an entire arrest population versus just those with a behavioral crisis. However, we studied repeat arrests in only one calendar year, so if an individual was arrested on December 28, 2015 and again on January 31, 2016, we did not count this situation as two arrests during calendar year 2016. Additionally, we were not able to account for jail or prison stays; therefore, individuals may not have had the same opportunity to be rearrested after the first arrest in 2016.

## Conclusion

This study found high rates of mental health and SUD diagnoses among the arrested population; however, the rate of repeat arrests is not driven solely by mental health conditions or substance use. Our findings suggest that linked clinical and criminal justice data systems may help better identify individuals at risk of repeat arrest and may help inform opportunities for interventions aimed at low-level offenders with behavioral health needs. Nevertheless, more data on clinical care utilization and other social factors related to the risk of repeat arrest are needed.

## Supplementary Information

**Additional file 1.**

## Data Availability

The authors do not have permission to share the data. Code is available by contacting the corresponding author.
