# Supplementary material for: Two-year prevalence rates of mental health and substance use disorder diagnoses among repeat arrestees
Source: Health Justice. 2021 Jan 7;9:2. doi: 10.1186/s40352-020-00126-2 (PMC7789256; doi:10.1186/s40352-020-00126-2)
Supplement: Supplementary file 1 — Additional file 1. [file 40352_2020_126_MOESM1_ESM.docx]

| **SUPPLEMENT A** | | |
| --- | --- | --- |
| Phenotype | ICD-9 Diagnosis Code | ICD-10 Diagnosis Code |
| Psychosis disorder diagnosis | 291.1; 292.9; 293.81-2; 295.1-9; 297.1; 297.3; 298.8; 298.9 | F20; F21; F06.0; F06.1; F06.2; F10.15; F10.150; F10.151; F10.159; F10.18; F10.25; F10.250; F10.251; F10.259; F10.95; F10.951; F10.959; F11.15; F11.150; F11.151; F11.159; F11.250; F11.251; F11.259; F11.95; F11.951; F11.959; F12.159; F12.25; F12.251; F12.259; F12.95; F12.951; F12.959; F13.15; F13.151; F13.159; F13.25; F13.251; F13.259; F13.95; F13.951; F13.959; F14.15; F14.151; F14.159; F14.25; F14.251; F14.259; F14.95; F14.951; F14.959; F15.15; F15.151; F15.159; F15.25; F15.251; F15.259; F15.95; F15.951; F15.959; F16.15; F16.151; F16.159; F16.25; F16.251; F16.259; F16.95; F16.950; F16.951; F16.959; F18.15; F18.151; F18.159; F18.25; F18.251; F18.259; F18.95; F18.951; F18.959; F19.15; F19.151; F19.159; F19.25; F19.251; F19.259; F19.95; F19.951; F19.959; F20.9; F22; F28; F29; F20.0; F20.1; F20.2; F20.3; F20.5; F24; F25; F25.8; F25.9; F20.81; F23; F25.0; F25.1 |
| Bipolar disorder diagnosis | 292.84;291.89; 296.00; 296.01; 296.02; 296.03; 296.04; 296.05; 296.06; 296.60; 296.61; 296.62; 296.63; 296.64; 296.65; 296.66; 296.90; 293.83; 296.40; 296.41; 296.42; 296.43; 296.44; 296.45; 296.46; 296.50; 296.51; 296.52; 296.53; 296.54; 296.55; 296.56; 296.7; 296.80; 296.89; 301.13 | F31;F311;F313;F317.1;F317.7;F318;F101.4;F102.4;F109.4;F111.4;F112.4;F119.4;F131.4;F132.4;F139.4;F141.4;F142.4;F149.4;F151.4;F152.4;F159.4;F161.4;F162.4; F169.4; F181.4; F182.4; F189.4; F191.4; F192.4; F199.4; F30; F30.1; F30.11; F30.12; F30.13; F30.2; F30.3; F30.8; F30.9; F31.6; F31.61; F31.62; F31.63; F31.64; F39; F06.33; F06.34; F31.11; F31.12; F31.13; F31.2; F31.31; F31.32; F31.4; F31.5; F31.73; F31.74; F31.75; F31.76; F31.81; F31.89; F31.9; F34.0 |
| Anxiety disorder diagnosis | 293.84; 300.09; 309.21; 313.23; 293.89; 300.22; 291.89; 292.89; 300.00; 300.01; 300.02; 300.21;300.23; 300.29 | F40; F40.01; F40.8; F40.9; F41; F06.4; F10.180; F10.280; F10.980; F12.180; F12.280; F12.980; F13.180; F13.280; F13.980; F14.180; F14.280; F14.980; F15.180; F15.280; F15.980; F16.180; F16.280; F16.980; F18.180; F18.280; F18.980; F19.180; F19.280; F19.980; F40.00; F40.1; F40.11;F40.2; F40.21; F40.218; F40.22; F40.228; F40.23; F40.230; F40.231; F40.232; F40.233; F40.24; F40.241; F40.242; F40.243; F40.248; F40.29; F40.291; F40.298; F41.0; F41.1; F41.8; F41.9; F93.0; F94.0; F40.02 |
| Personality disorder diagnosis | 301.89; 310.1; 301.22; 301.0; 301.20; 301.4; 301.50; 301.6; 301.7; 301.81; 301.82; 301.83; 301.9 | F07.0; F21; F60.0; F60.1; F60.2; F60.3; F60.4; F60.5; F60.6; F60.7; F60.81; F60.89; F60.9 |
| Disruptive disorder diagnosis | 301.7; 312.81; 312.82; 312.89; 312.30; 312.31; 312.39; 312.8; 312.32; 312.33; 312.34; 312.9; 313.81 | F91; F60.2; F63.1; F63.2; F63.81; F91.8; F91.9; F63; F63.8; F63.89; F63.9; F91.1; F91.2; F91.3 |
| Substance Use Disorder | 304.7;304.71; 304.72; 304.73; 292.9; 292.0; 292.1; 292.11; 292.12; 292.2; 292.8; 292.81; 292.82; 292.83; 292.84; 292.85; 292.89; 304.1; 304.11; 304.12; 304.13; 304.2; 304.21; 304.22; 304.23; 304.3; 304.31; 304.32; 304.33; 304.4; 304.41; 304.42; 304.43; 304.5; 304.51; 304.52; 304.53; 304.6; 304.61; 304.62; 304.63; 304.8; 304.81; 304.82; 304.83; 304.9; 304.91; 304.92; 304.93; 305.2; 305.21; 305.22; 305.23; 305.3; 305.31; 305.32; 305.33; 305.4; 305.41; 305.42; 305.43; 305.6; 305.61; 305.62; 305.63; 305.7; 305.71; 305.72; 305.73; 305.8; 305.81; 305.82; 305.83; 305.9; 305.91; 305.92; 305.93; 648.3; 648.31; 648.32; 648.33; 648.34; 304.0; 304.01; 304.02; 304.03; 305.5; 305.51; 305.52; 305.53 | F121.20; F12.121; F12.122; F12.129; F12.15; F12.150; F12.151; F12.159; F12.18; F12.180; F12.188; F12.19; F12.2; F12.20; F12.21; F12.22; F12.220; F12.221; F12.222; F12.229; F12.23; F12.25; F12.250; F12.251; F12.259; F12.28; F12.280; F12.288; F12.29; F12.9; F12.90; F12.92; F12.920; F12.921; F12.922; F12.929; F12.93; F12.95; F12.950; F12.951; F12.959; F12.98; F12.980; F12.988; F12.99; F13; F13.1; F13.10; F13.11; F13.12; F13.120; F13.121; F131.29; F13.14; F13.15; F13.150; F13.151; F13.159; F13.18; F13.180; F13.181; F13.182; F13.188; F13.19; F13.2; F13.20; F13.21; F13.22; F13.220; F132.21; F13.229; F13.23; F13.230; F13.231; F13.232; F13.239; F13.24; F13.25; F13.250; F13.251; F13.259; F13.26; F13.27; F13.28; F13.280; F13.281;F13.282; F13.288; F13.29; F13.9; F13.90; F13.92; F13.920; F13.921; F13.929; F13.93; F13.930; F13.931; F13.932; F13.939; F13.94; F13.95; F13.950; F13.951; F13.959; F13.96; F13.97; F13.98; F139.80; F13.981; F13.982; F13.988; F13.99; F14; F14.1; F14.10; F14.11; F14.12; F14.120; F14.121; F14.122;F14.129; F14.14; F14.15; F14.150; F14.151; F14.159; F14.18; F14.180; F14.181; F14.182; F14.188; F14.19; F14.2; F14.20; F14.21; F14.22; F14.220; F14.221; F14.222; F14.229; F14.23; F14.24; F14.25; F14.250; F14.251; F14.259; F14.28; F14.280; F14.281; F14.282; F14.288; F14.29; F149; F149.0; F14.92; F149.20; F14.921; F14.922; F14.929; F14.94; F14.95; F14.950; F14.951; F14.959; F14.98; F14.980; F14.981; F14.982; F14.988; F14.99; F15; F15.1; F15.10; F15.11; F15.12; F15.120; F15.121; F15.122; F15.129; F15.14; F15.15; F15.150; F15.151; F15.159; F15.18; F15.180; F15.181; F15.182;F15.188; F15.19; F15.2; F15.20; F15.21; F15.22; F15.220; F15.221; F15.222; F15.229; F15.23; F15.24; F15.25; F15.250; F15.251; F15.259; F15.28; F15.280; F15.281; F15.282; F15.288; F15.29; F15.9; F15.90; F15.92; F15.920; F15.921; F15.922; F15.929; F15.93; F15.94; F15.95; F15.950; F15.951; F15.959; F15.98; F15.980; F15.981; F15.982; F15.988; F15.99; F16; F16.1; F16.10; F16.11; F16.12; F16.120; F16.121; F16.122; F16.129; F16.14; F1615; F16.150; F16.151; F16.159; F16.18; F16.180; F16.183; F16.188; F16.19; F16.2; F16.20; F16.21; F16.22; F16.220; F16.221; F16.229; F16.24; F16.25; F16.250; F16.251; F16.259; F16.28; F16.280; F16.283; F16.288; F16.29; F16.9; F16.90; F16.92; F16.920; F16.921; F16.929; F16.94; F16.95; F16.950; F16.951; F16.959; F16.98; F16.980; F16.983; F16.988; F16.99; F18; F18.1; F18.10; F18.11; F18.12; F18.120; F18.121; F18.129; F18.14; F18.15; F18.150; F18.151; F18.159; F18.17; F18.18; F18.180; F18.188; F18.19; F18.2; F18.20; F18.21; F18.22; F18.220; F18.221; F18.229; F18.24; F18.25; F18.250; F18.251; F18.259; F18.27; F18.28; F18.280; F18.288; F18.29; F18.9; F18.90; F18.92; F18.920; F18.921; F18.929; F18.94; F18.95; F18.950; F18.951; F18.959; F18.97; F18.98; F18.980; F18.988; F18.99; F19; F19.1; F19.10; F19.11; F19.12; F19.120; F19.121; F19.122; F19.129; F19.14; F19.15; F19.150; F191.51; F1.959; F19.16; F19.17; F19.18; F19.180; F19.181; F19.182; F19.188; F19.19; F19.2; F19.20; F19.21; F19.22; F19.220; F19.221; F19.222; F19.229; F19.23; F19.230; F19.231; F19.232; F19.239; F19.24; F19.25; F19.250; F19.251; F19.259; F19.26; F19.27; F19.28; F19.280; F19.281; F19.282; F19.288; F19.29; F19.9; F19.90; F19.92; F19.920; F19.921; F19.922; F19.929; F19.93; F19.930; F19.931; F19.932; F19.939; F19.94; F19.95; F19.950; F19.951; F19.959; F19.96; F19.97; F19.98; F19.980; F19.981; F19.982; F19.988; F19.99; F55; F55.8; O99.32; O99.320; O99.321; O99322;O99.323; O99.324; O99.325 |
|  |  |  |
